# Sunflower-Derived Pectin Oligogalacturonides Promote Defecation in Mice

**DOI:** 10.3390/foods15152625

**Published:** 2026-07-27

**Authors:** Hongming Gu, Xian Qu, Xiaotong Sheng, Xuran Wang, Yuhan Cai, Jie Geng, Liangnan Cui, Kevin H. Mayo, Yifa Zhou, Hairong Cheng, Guihua Tai

**Affiliations:** 1 Engineering Research Center of Glycoconjugates, Ministry of Education, Jilin Provincial Key Laboratory of Chemistry and Biology of Changbai Mountain Natural Drugs, School of Life Sciences, Northeast Normal University, Changchun 130024, China; guhm154@nenu.edu.cn (H.G.); quxian2025@xmu.edu.cn (X.Q.); shengxt371@nenu.edu.cn (X.S.); wangxuran975@nenu.edu.cn (X.W.); caiyuhan123@nenu.edu.cn (Y.C.); gengj477@nenu.edu.cn (J.G.); cuiln802@nenu.edu.cn (L.C.); zhouyf383@nenu.edu.cn (Y.Z.); 2 Department of Biochemistry, Molecular Biology & Biophysics, 6-155 Jackson Hall, University of Minnesota, 321 Church Street, Minneapolis, MN 55455, USA; mayox001@umn.edu

**Keywords:** sunflower pectin, enzyme transformation, oligosaccharides, constipation, defecation, gut microbiota, SCFA

## Abstract

Sunflower pectin is widely used as a food additive due to its gelling and stabilizing properties. However, its bioactive potential remains poorly understood. Here, we explored its effect on defecation. Using a diphenoxylate-induced constipation mouse model, we found that native sunflower pectins did not significantly promote defecation. Subsequently, we investigated whether they could be activated via biotransformation. For this, we enzymatically hydrolyzed the pectin and produced a number of fragments that covered a wide range of molecular weights (0.5 to 30 kDa) in two esterification states (0% and 10% methyl esterification). Interestingly, all fragments exhibited defecation-promoting effects. Moreover, the effect progressively increased as the molecular weight decreased, a trend that was observed with both esterified and non-esterified fragments. Comparative studies identified E3 as the most effective fraction. E3 also displayed a prebiotic effect in the gut of model mice. Structural analysis indicated that E3 comprises oligogalacturonides with degrees of polymerization from one to six, mainly DP3 and DP4. Overall, our study reveals an inverse correlation of molecular weight with defecation-promoting activities, and offers a simple and scalable approach to convert ineffective pectin into effective oligogalacturonides that have the potential to become functional food ingredients.

## 1. Introduction

Sunflower pectin, extracted from sunflower heads that are generated during oil processing, is a structural heteropolysaccharide whose backbone consists primarily of linear α-1,4-linked galacturonic acid (GalA) residues [1,2,3]. This pectin is predominantly minimally methyl-esterified, which distinguishes it from common fruit-derived pectins [4]. As a natural food additive, it is widely used in jams, jellies, beverages, and confectioneries due to its emulsifying, thickening, gelling, and stabilizing properties [5,6]. However, its potential as a bioactive functional food ingredient for gut health has not been fully recognized.

To date, only a limited number of studies have reported some biological activities of sunflower pectin. For instance, in vitro fermentation assays have demonstrated its prebiotic potential in promoting beneficial gut bacteria and short-chain fatty acid production [7]. Its DPPH and ABTS radical-scavenging activities have also been reported [2]. Ultrasound-extracted sunflower pectin has been shown to ameliorate acute inflammation and improve intestinal mucosal barrier function [8]. Nevertheless, the current understanding of sunflower pectin bioactivities remains limited. The present study evaluates its effect on constipation.

Constipation is a gastrointestinal disorder that affects patients’ daily lives [9]. Current pharmacological treatments include osmotic laxatives, stimulant laxatives, secretagogues, and prokinetic agents [10,11]. These medications are often associated with adverse effects and tolerance upon prolonged use, which has prompted a search for alternative or complementary approaches [12,13]. In this regard, dietary fibers have attracted much attention due to their low toxicity and promising laxative effects [14,15]. Pectin is one of the most abundant soluble fibers and has been studied for its potential gut health benefits [16]. Clinical trials in adults with chronic slow-transit constipation have shown that apple pectin supplementation shortens colonic transit time and improves symptoms [17,18]. Similar defecation-promoting effects have been reported for pectin-containing mixtures, such as prune juice [19]. In animal models, pectins from burdock root, citrus peel, and orange have also shown defecation-promoting effects [20,21,22,23]. Despite these favorable findings, the defecation effectiveness of sunflower head-derived pectin has remained largely unexplored, and the structural determinants of such activity have not been investigated.

In the present study, we initially investigated three native sunflower pectins and their de-esterified counterparts, and we found that none of them significantly alleviated constipation. We then focused on transforming these ineffective pectins to effective substances. Employing two degradation strategies, we produced a series of pectin fragments and evaluated their efficacy in a mouse model of constipation. Our study revealed an inverse correlation between molecular weight (Mw) and the constipation-alleviating effect, and we established a simple method to produce highly active pectic oligomers. These findings highlight the potential of sunflower-derived oligogalacturonides as a prebiotic functional food ingredient for constipation relief.

## 2. Materials and Methods

### 2.1. Materials and Reagents

Sunflower heads were collected in Baicheng City, Jilin Province, China. Pectin sample P130 was obtained from Inner Mongolia CONSTAN Biotechnology Co., Ltd. (Bayannur, China). Pectinase was purchased from Shandong Lonct Enzymes Co., Ltd. (Linyi, China). This commercial multi-enzyme complex, fermented and refined from *Aspergillus niger*, contains pectin methyl-esterase (PE), polygalacturonase (PG), pectin methyl-galacturonase (PMG), pectate lyase (PGL), and pectin lyase (PL), which synergistically hydrolyze pectin substrates. Diphenoxylate was supplied by Changzhou Kangpu Pharmaceutical Co., Ltd. (Changzhou, China). Activated carbon was purchased from Tianjin Huadong Reagent Factory (Tianjin, China). Acacia gum was purchased from Aladdin Biochemical Technology Co., Ltd. (Shanghai, China). All reagents used in the preparation of sunflower pectin fragments were of food grade. All reagents used in the structural analysis were of analytical or HPLC grade.

### 2.2. Preparation of Pectin Samples

#### 2.2.1. Pectin Sample P30

Pectin was extracted from dried sunflower heads as previously reported [24], with minor modifications. Briefly, sunflower heads were immersed in 0.1% (*w*/*v*) oxalic acid solution (solid-to-liquid ratio of 1:16 g/mL) and heated at 100 °C for 2 h under reflux. The supernatant was collected by filtration through four layers of gauze, and the solid residue was re-extracted once using the same conditions. The combined filtrates were centrifuged (4000× *g*, 15 min) to remove insoluble material, and the supernatant was concentrated under reduced pressure. Pectin was precipitated by adding ethanol to a final concentration of 60% (*v*/*v*), and the precipitate was washed sequentially with 95% (*v*/*v*) and anhydrous ethanol to yield the crude pectin fraction. This fraction was purified on a preparative Sepharose CL-6B column (3.0 cm × 90 cm, Cytiva, Uppsala, Sweden) eluted with 0.15 M NaCl at 0.4 mL/min to obtain P30. The yield (%) was calculated as shown in Equation (1):

(1) $$\begin{array}{c} Yield\left(\%\right)\;=\;\frac{m_{\mathrm{isolated}\;\mathrm{pectin}}}{m_{\mathrm{dry}\;\mathrm{sunflower}\;\mathrm{head}}}\;\times \;100 \end{array}$$

where *m*_isolated pectin_ represents the dry weight of the recovered pectin fraction, and *m*_dry sunflower head_ is the dry weight of the sunflower head prior to extraction.

#### 2.2.2. Pectin Sample P75

The same procedure used for P30 production was employed to prepare P75, albeit with a different concentration of oxalic acid, reaction temperature, and duration, i.e., 0.2% oxalic acid and 80 °C for 1 h.

#### 2.2.3. Pectin Sample P130

Pectin sample P130 (pharmaceutical grade, model: CSP202, batch: FP200096) was commercially obtained from Inner Mongolia CONSTAN Biotechnology Co., Ltd. (Bayan Nur City, China) and used as received.

#### 2.2.4. Pectin Samples P30D, P75D, and P130D

Native pectin samples P30, P75, and P130 were de-esterified. Specifically, each pectin was treated with 0.1 M NaOH at 4 °C for 7 h, and the mixture was neutralized using hydrochloric acid, dialyzed and desalted using a 3 kDa Mw cut-off hollow fiber ultrafiltration system, concentrated by vacuum distillation, and finally lyophilized. The resulting de-esterified samples were called P30D, P75D, and P130D. The yield (%) was calculated as shown in Equation (2):

(2) $$\begin{array}{c} Yield\left(\%\right)\;=\;\frac{m_{\mathrm{de}-\mathrm{esterified}\;\mathrm{pectin}}}{m_{\mathrm{esterified}\;\mathrm{pectin}}}\;\times \;100 \end{array}$$

where *m*_de-esterified pectin_ represents the dry weight of recovered P30D, P75D, or P130D; and *m*_esterified pectin_ is the dry weight of P30, P75, or P130.

### 2.3. Preparation of De-Esterified Pectin Fragments from P130D

#### 2.3.1. Screening for Hydrolysis Conditions

Time-dependent hydrolysis was done to optimize the reaction time. For this, P130D (50 mg) was dissolved in 5 mL of 20 mM sodium acetate–acetic acid buffer (pH 4.0) to a final concentration of 10 mg/mL. After pre-incubation at 50 °C for 30 min, the reaction was initiated by adding pectinase at an enzyme-to-substrate ratio of 0.352 mU/g and maintained at 50 °C. Aliquots (200 μL) were collected at 4, 8, 12, 18, 36, and 108 h and processed for Mw analysis as described in Section 2.6.

#### 2.3.2. Production of Fragments DE4, DE8, DE12, and DE18

Based on the kinetic profile, four hydrolysis times (4, 8, 12, and 18 h) were selected for scale-up production. For this procedure, P130D (100 g) was dissolved in 20 mM sodium acetate–acetic acid buffer (pH 4.0), and pectinase was added at a ratio of 0.352 mU/g, with reactions carried out at 50 °C before heat inactivation. Reaction mixtures were desalted using a 10 kDa or 1 kDa hollow fiber ultrafiltration system (as applicable), concentrated by rotary evaporation, and then lyophilized. This procedure yielded fragments designated DE4, DE8, DE12, and DE18, corresponding to the four hydrolysis reaction times. The yield (%) was calculated as shown in Equation (3):

(3) $$\begin{array}{c} Yield\left(\%\right)\;=\;\frac{m_{\mathrm{fragment}}}{m_{P130D}}\;\times \;100 \end{array}$$

where *m*_fragment_ represents the dry weight of recovered DE4, DE8, DE12, or DE18; and *m*_P130D_ is the dry weight of P130D.

### 2.4. Preparation of Pectin Fragments from P130

#### 2.4.1. Screening for Enzymatic Hydrolysis

To determine the optimal enzyme concentration, P130 was dissolved in distilled water (10 mg/mL) at a natural pH of ~2.5. Pectinase was added to the solution, and the mixture was incubated at 50 °C for 10 h. Pectinase-to-substrate ratios ranged from 0.22 to 7.04 mU/g. Upon completion of the hydrolysis reaction, aliquots (200 μL) were collected from each reaction mixture.

Each aliquot (200 μL) was boiled for 5 min to terminate the reaction and centrifuged at 12,000 rpm for 5 min. The resulting supernatant was collected, diluted with distilled water to a final pectin concentration of 5 mg/mL, and then filtered through a 0.22 μm membrane filter prior to Mw analysis.

#### 2.4.2. Production of Fragments E1, E2, and E3

Following optimization of pectinase hydrolysis, we scaled-up production. Briefly, 100 g of P130 was dissolved in 10 L of ddH_2_O preheated to 50 °C (final concentration 10 mg/mL, pH ~ 2.5). Reactions were run at 50 °C for 10 h using three enzyme-to-substrate ratios: 0.88, 1.76, and 3.52 mU/g. Pectinase was then inactivated by heating at 80 °C for 1 h. Hydrolysates were uniformly stirred, discharged from the reactor, and then concentrated by vacuum distillation. Insoluble material was removed by centrifugation (6000 rpm, 20 min, 25 °C), and the resulting supernatants were lyophilized to obtain fragments E1, E2, and E3, which corresponded to the three enzyme ratios. The yield (%) was calculated as shown in Equation (4):

(4) $$\begin{array}{c} Yield\left(\%\right)\;=\;\frac{m_{\mathrm{fragment}}}{m_{P130}}\;\times \;100 \end{array}$$

where *m*_fragment_ represents the dry weight of the recovered E1, E2 or E3; and *m*_P130_ is the dry weight of P130.

### 2.5. Monosaccharide Composition Analysis

The monosaccharide composition of pectin samples was determined using high-performance liquid chromatography (HPLC) following pre-column derivatization with 1-phenyl-3-methyl-5-pyrazolone (PMP) [25].

### 2.6. Molecular Weight Determination

The weight-average Mw of samples was determined using high-performance gel permeation chromatography (HPGPC) on a TSK-gel G-3000 PWXL column (7.8 mm × 300 mm, TOSOH, Tokyo, Japan) and monitored with a refractive index detector [26].

### 2.7. Determination of the Degree of Methyl Esterification

For complete ionization of carboxyl groups (-COOH) to eliminate the influence of organic acids when determining the degree of methyl esterification (DM), we first dissolved a pectin sample in ddH_2_O, adjusted the pH to 6~7 with 0.1 M NaOH, and then lyophilized the sample. A total of 1 to 2 mg of the lyophilized sample was mixed with an appropriate amount of KBr, ground into a powder, and pressed into a pellet. The DM of these pectin samples was determined using FT-IR spectroscopy (PerkinElmer, Waltham, MA, USA) over the range of 4000–400 cm^−1^. Absorbance bands at ~1740 cm^−1^ and ~1630 cm^−1^ that correspond to esterified and free carbonyl groups, respectively, were used for DM calculation (Equation (5)) as the ratio of the peak area at 1740 cm^−1^ to the sum of the peak areas at 1740 cm^−1^ and 1630 cm^−1^ [27].

(5) $$\begin{array}{c} DM\left(\%\right)=\left[\frac{A_{1740}}{A_{1740}+A_{1630}}\right]\times 100 \end{array}$$

### 2.8. Structural Analysis of E3 by UPLC-ESI-MS

The composition of E3 was analyzed using an Acquity UPLC system (Waters, Milford, MA, USA) coupled with an Amazon Speed ETD mass spectrometer (Bruker, Bremen, Germany). Chromatographic separation was performed on a Waters BEH Amide column (1.7 μm, 2.1 mm × 150 mm, Waters, Milford, MA, USA) and maintained at 35 °C. The sample concentration was 0.5 mg/mL, and the injection volume was 10 μL. The mobile phase consisted of solvent A (20% acetonitrile), solvent B (80% acetonitrile), and solvent C (0.2 M Formate–Ammonium Formate buffer, pH 3.0). A gradient elution program was run over 60 min at a flow rate of 0.3 mL/min.

Mass spectrometric analysis was carried out on an Amazon Speed ETD instrument (Bruker, Germany) operated in the negative ion mode. Electrospray ionization (ESI) parameters were set as follows: capillary voltage, 4.0 kV; end plate offset, 300 V; capillary temperature, 200 °C; nebulizer gas pressure, 29 psi; and dry gas flow rate, 8 L/min. Full-scan spectra were acquired across the m/z range of 50–2000. Data were processed using the Trap-control software (v7.2) [28].

### 2.9. Experimental Animals

All animal studies were conducted with approval from the Animal Research Ethics Committee of Northeast Normal University (protocol code 202602001, date of approval: 15 January 2026) and performed in accordance with the established guidelines. Specific pathogen-free female ICR mice (5 weeks old, 20 ± 2 g) were housed under controlled conditions and acclimatized for 7 days. Mice were housed in individually ventilated cages (IVC) on autoclaved bedding under controlled conditions (temperature 22 ± 2 °C, relative humidity 50 ± 10%, 12 h light/dark cycle, lights on at 7:00 AM). Mice were provided ad libitum access to a standard commercial rodent chow (SPF-grade, supplied by Jiangsu Xietong Pharmaceutical Bio-Engineering Co., Ltd., Nanjing, China) containing 5% crude fiber, 20% crude protein, and 4% crude fat. Water was provided ad libitum throughout the experiment. The sample size (*n* = 8 per group) was determined by power analysis using the G*Power software (version 3.1). Based on the effective size observed in our pilot experiments (GI transit ratio, Cohen’s *f* = 0.45, a large effect), with a significance of *α* = 0.05 and a desired statistical power of 1-*β* = 0.8, a minimum of 7 animals per group were required. To account for potential attrition or data exclusion, each group consisted of 8 animals. Mice were randomly divided into groups (*n* = 8): a normal control (C), a model control treated with diphenoxylate (Dip), and low- (0.2 g/kg BW) and high-dose (1 g/kg BW) pectin-treated groups. All groups received their corresponding treatments by daily oral gavage for 14 consecutive days. The C and Dip groups received an equivalent volume of distilled water.

### 2.10. Assessment of the Constipation-Alleviating Effect

#### 2.10.1. Gastrointestinal Transit Ratio

After the 14-day treatment, fasted mice were orally administered 0.025% diphenoxylate. Thirty minutes later, a 10% activated carbon suspension was given. Mice were sacrificed 25 min after carbon administration. The gastrointestinal (GI) transit ratio (%) = the distance traveled by activated carbon meal/the total small intestinal length × 100% [29].

#### 2.10.2. Black Fecal Parameters

Following treatment and 16 h fasting, mice were given 0.1% diphenoxylate and, 30 min later, a 10% activated carbon suspension. They were then individually placed in clean cages and observed for 5 h. The times to release of the first black stool, as well as the total number and weight of black feces excreted, were recorded [30].

### 2.11. GC Analysis of Short-Chain Fatty Acids

Approximately 100 mg of cecal content was homogenized in distilled water, vortexed for 2 h, and then centrifuged. The supernatant was acidified with 50% sulfuric acid and extracted with dichloromethane (DCM). After centrifugation, the lower DCM layer was filtered through a 0.22 μm organic membrane and analyzed by GC. Blank and standard samples were processed in parallel for quality control [31].

### 2.12. 16S rRNA Sequencing

Cecal content samples were flash-frozen in liquid nitrogen and stored at −80 °C. Total genomic DNA was extracted, and the V4 region of the 16S rRNA gene was amplified with barcoded primers 515F/806R. PCR products were purified with magnetic beads, mixed at equal concentration ratios, and libraries were prepared with index addition. Libraries were quantified by Qubit (Thermo Fisher Scientific, Waltham, MA, USA) and real-time PCR, size-checked by bioanalyzer, and sequenced on an Illumina NovaSeq 6000 platform (Illumina, San Diego, CA, USA). Paired-end reads were demultiplexed and trimmed of barcodes/primers, then merged using FLASH v1.2.11 [32] to obtain raw tags. Quality filtering was performed with fastp v0.23.1 [33] to generate clean tags. Chimeras were detected by comparison to the Silva database (for 16S rRNA) and removed using vsearch v2.16.0 [34], yielding effective tags. ASVs (amplicon sequence variants) were obtained by denoising with DADA2 (default) in QIIME2 (v2021.4) [35]. Species annotation was performed with QIIME2 against the Silva database; for non-standard regions, the Micro NT database (a subset of NCBI NT) was used via BLAST (v2.13.0) followed by LCA classification, ignoring unclassified hits. Remaining unannotated sequences were supplemented using NCBI dmp files.

### 2.13. Statistical Analysis

All statistical analyses were performed using GraphPad Prism 11.0.2 and Origin 8.5. Results are expressed as the mean ± SD. The normalization of data distribution was assessed using the Shapiro–Wilk test, and the homogeneity of variances was verified by using Levene’s test. Since all datasets satisfied the assumptions of normality and homoscedasticity (*p* > 0.05 for all groups), differences between multiple groups were analyzed by one-way ANOVA, followed by Tukey’s post hoc test. Significance levels were denoted as follows: * *p* < 0.05; ** *p* < 0.01; *** *p* < 0.001; and **** *p* < 0.0001. Standardized between-group effect sizes were quantified using Cohen’s *d*. Cohen’s *d* values and 95% confidence intervals (95% CIs) were calculated based on group means, standard deviations, and sample sizes using an effect size calculator (https://acdelre.com/apps/compute_es/, accessed on 12 July 2026) (Table S1). According to Cohen’s conventions, the standard benchmarks for interpreting *d* values are as follows: small effect (*d* ≈ 0.2), medium effect (*d* ≈ 0.5), and large effect (*d* ≥ 0.8).

## 3. Results and Discussion

### 3.1. Preparation and Characterization of Sunflower Pectin Polymers

This study was initiated by preparing a series of pectin polymers from sunflower heads. From this preparation, we extracted two pectin fractions (P30 and P75) and obtained a third commercial sample (P130). De-esterified fractions (P30D, P75D, and P130D) were generated by treating pectins P30, P75, and P130 with 0.1 M NaOH at 4 °C for 7 h, followed by neutralization, dialysis, and lyophilization.

**Figure 1:**
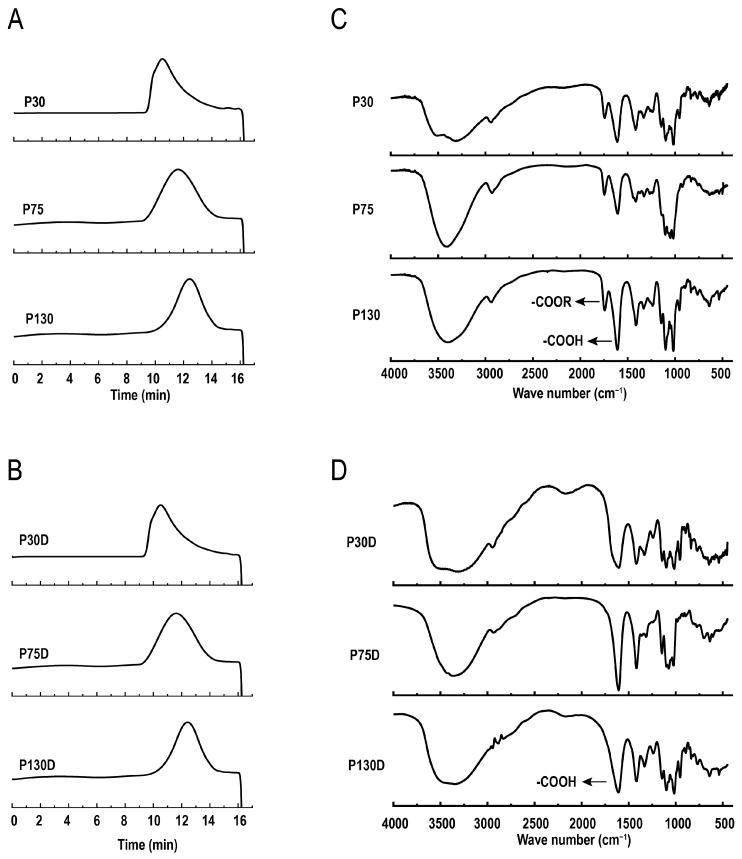
Preparation and characterization of sunflower pectin polymers P30, P75, and P130. (**A**) HPGPC elution profiles of P30, P75, and P130 on TSK gel G3000. (**B**) HPGPC elution profiles of P30D, P75D, and P130D on TSK gel G3000. (**C**) FT-IR spectra of the native pectins P30, P75, and P130 (**D**) FT-IR spectra of the de-esterified pectins P30D, P75D, and P130D.

HPGPC analysis determined the Mws of P30, P75, and P130 to be ~30, 75, and 130 kDa, respectively (Figure 1A). The de-esterified pectins exhibited similar Mw distributions to their native forms (Figure 1B). Monosaccharide composition showed that all six pectin fractions were HG-rich, with GalA content exceeding 85.2% (Table 1).

The degree of methyl-esterification (DM) was quantitatively assessed using FT-IR spectroscopy by analyzing the characteristic absorption bands of carboxyl groups (Figure 1C,D). In the spectra of native pectins (P30, P75, and P130), two major distinct peaks were observed: a prominent band at approximately 1740 cm^−1^, assigned to the C=O stretching vibration of esterified carboxyl groups (–COOR); and another strong band at around 1630 cm^−1^, corresponding to the asymmetric stretching vibration of free carboxylate anions (–COOH). To accurately determine the DM values, we performed baseline correction and calculated the integrated peak areas for both bands. The DM was then calculated using Equation (5). This quantification revealed DM values of approximately 25%, 30%, and 20% for P30, P75, and P130, respectively (Table 1), which are consistent with previously reported values for sunflower pectins [36]. De-esterified variants (P30D, P75D, P130D) lacked the peak at ~1740 cm^−1^, thus confirming the successful removal of methyl ester groups (Figure 1D, Table 1).

These polymers served as a crucial first step in screening for pectin-based ingredients with bioactive potential and guided our subsequent bioconversion strategy.

### 3.2. Effect of Sunflower Pectin Polymers on Constipation in Mice

**Figure 2:**
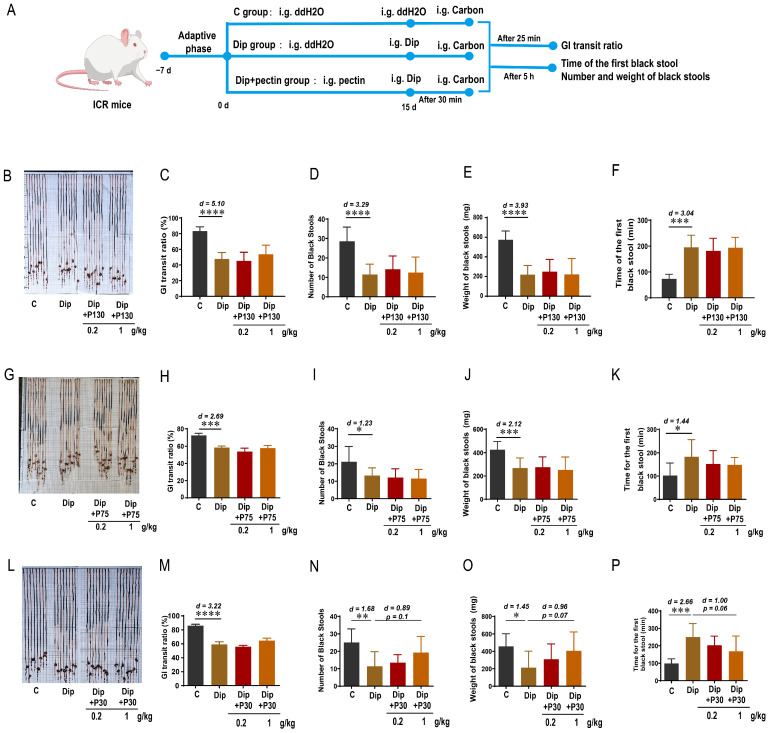
Assessment of the constipation-alleviating effects of sunflower pectin polymers P30, P75, and P130. (**A**) Scheme of animal experiments used in this study. (**B**–**F**). Effects of high-dose (1 g/kg) and low-dose (0.2 g/kg) P130: a picture of the small intestine (**B**), the GI transit ratio (**C**), the number and weight of black stools within 5 h (**D**,**E**), and the time of the first black stool (**F**). (**G**–**K**). Effects of high-dose and low-dose P75: a picture of the small intestine (**G**), the GI transit ratio (**H**), the number and weight of black stools within 5 h (**I**,**J**), and the time of the first black stool (**K**). (**L**–**P**) Effects of high-dose and low-dose P30: a picture of the small intestine (**L**), the GI transit ratio (**M**), the number and weight of black stools within 5 h (**N**,**O**), and the time of the first black stool (**P**). C: control group. Dip: diphenoxylate. Carbon: activated carbon. GI: gastrointestinal. Data are shown as the mean ± SD (*n* = 8). * *p* < 0.05, ** *p* < 0.01, *** *p* < 0.001, **** *p* < 0.0001. Effect sizes were interpreted according to Cohen’s conventions (*d* ≈ 0.2, 0.5, and ≥0.8 for small, medium, and large effects, respectively).

We assessed the efficacy of pectin and its fragments against constipation using a diphenoxylate-induced constipation mouse model. As outlined in Figure 2A, mice were divided into C, Dip, and pectin groups. Mice were provided water ad libitum (for C and Dip groups) or water-based pectic samples (for pectin groups) for two weeks and then given diphenoxylate (for Dip and pectin groups) followed by a carbon diet for intestinal labeling. Mice were sacrificed and analyzed for transit time through the small intestine by measuring the GI transit ratio, which reflects small intestinal propulsion. The transit time through the entire intestine was measured from the time of appearance of the first black stool and the total number and weight of black stools within 5 h.

The reliability of this model was verified by comparison with the control group. In this regard, the model group’s GI transit time and total number and weight of black feces within 5 h were significantly reduced, whereas the time that the first black stool appeared was significantly increased compared to the control group (Figure 2).

Initially, we evaluated the effects of the six pectin polymers in the diphenoxylate-induced constipated mouse model at doses of 0.2 and 1 g/kg. P130 and P75 showed no significant effects at either dosage (Figure 2B–K). At the high dose (1 g/kg), P30 modestly reduced the time of appearance of the first black stool and increased fecal output (Figure 2L–P).

Similar to the trend observed for their native counterparts, de-esterified pectins P130D and P75D remained ineffective (Figure S1A–J), whereas P30D showed some activity at the high dose (1 g/kg), with comparable effects to that of P30 in terms of reducing the appearance time for the first black stool and increasing fecal output (Figure S1K–O).

In both instances, pectins with relatively lower Mws (30 kDa) displayed some albeit insignificant activity, whereas those with relatively higher Mws (75 and 130 kDa) showed no activity. These results suggest that the size of pectin is an important factor in alleviating constipation. Because of this, we speculated that reducing the size of pectin may enhance its efficacy or convert it from a non-effective pectin to an effective one. Based on this hypothesis, we carried out a series of degradation processes to convert the non-effective pectins into pectic fragments with Mw ≤ 30 kDa, as shown in the following sections.

### 3.3. Degradation of P130D into Fragments DE4, DE8, DE12, and DE18

Having determined that native sunflower pectins have no or only minimal defecation-promoting activity, we enzymatically hydrolyzed P130D, a de-esterified pectin, to generate a series of fragments for assessing structure–activity relationships (Mw vis-à-vis activity). The fragments generated from P130D would eliminate the confounding effect of methyl ester groups, thereby allowing an unambiguous assessment of the impact of Mw on this activity. To screen the hydrolysis conditions, P130D was treated with pectinase at an enzyme-to-substrate ratio of 0.352 mU/g at 50 °C for 4, 8, 12, 18, 36, and 108 h (Figure S2A). HPGPC analysis showed the expected decrease in Mw with increasing hydrolysis time (Figure S2B). After 4 h treatment, hydrolysis was clearly observed with the primary products eluted at 11.5 min. After 8 h treatment, hydrolysis continued, and the main products were eluted at 12.5 min. After 12 h treatment, the products were further degraded, and a group of peaks appeared at 14–15 min. After 18 h treatment, nearly all products were eluted at 14–15 min. After 36 h treatment, degradation was intensified. Extending the hydrolysis time to 108 h resulted in a single peak eluted at 15.2 min, which was the elution time for monosaccharide GalA.

**Figure 3:**
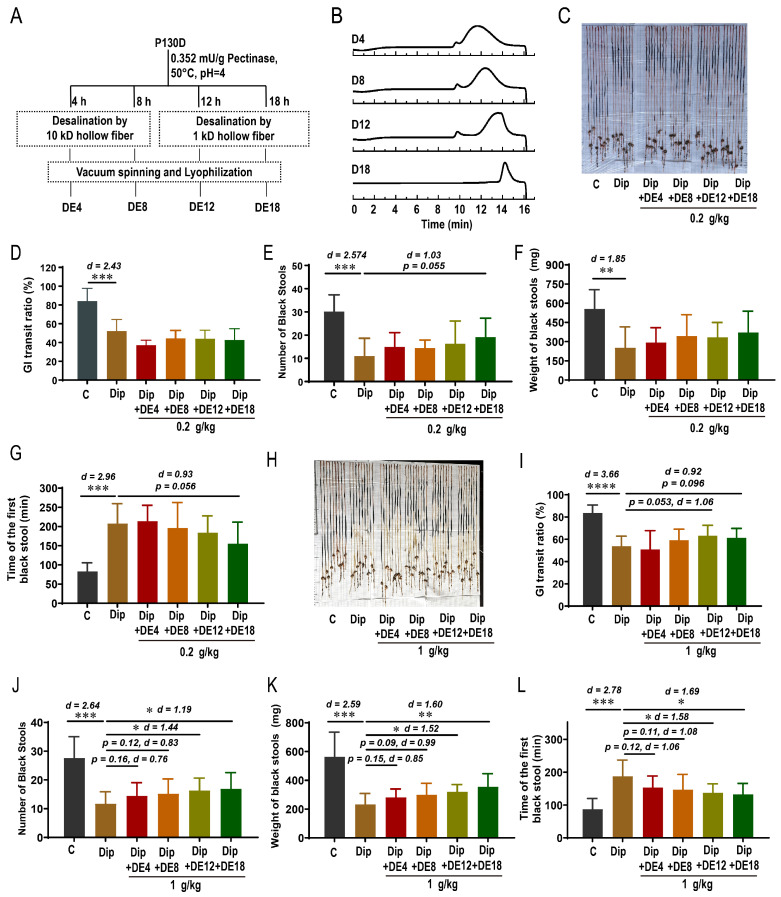
Generation and defecation-promoting effects of de-esterified pectin fragments DE4, DE8, DE12, and DE18 from P130D. (**A**) Scheme for preparation of DE series fragments. (**B**) Mw distribution profiles of DE series fragments. (**C**–**G**) Effects of low-dose (0.2 g/kg) fraction DEs: a picture of the small intestine (**C**), the GI transit ratio (**D**), the number and weight of black stools within 5 h (**E**,**F**), and the time of appearance of the first black stool (**G**). (**H**–**L**) Effects of high-dose (1 g/kg) fraction DEs: a picture of the small intestine (**H**), the GI transit ratio (**I**), the number and weight of black stools within 5 h (**J**,**K**), and the time of appearance of the first black stool (**L**). C: control group. Dip: diphenoxylate. GI: gastrointestinal. Data are shown as the mean ± SD (*n* = 8). * *p* < 0.05, ** *p* < 0.01, *** *p* < 0.001, **** *p* < 0.0001. Effect sizes were interpreted according to Cohen’s conventions (*d* ≈ 0.2, 0.5, and ≥0.8 for small, medium, and large effects, respectively).

Based on the kinetic profile, we selected four hydrolysis times (4, 8, 12, and 18 h) for scale-up production (Figure 3A) of the obtained fractions DE4 (78.4%), DE8 (48.9%), DE12 (20.1%), and DE18 (9.0%) (Table 1), respectively. As detected by HPGPC, their Mws were ~30, 15, 4, and <1 kDa, respectively (Figure 3B, Table 1). Monosaccharide compositions of DE4, DE8, DE12, and DE18 were similar to each other and to their parent pectin P130D (Table 1). As expected, all DEs showed the absence of esterification (Table 1, Figure S3A).

### 3.4. Effect of Fractions DE4, DE8, DE12, and DE18 on Constipation

Next, we assessed the efficacy of fractions DE4, DE8, DE12, and DE18. At 0.2 g/kg BW (Figure 3C–G), none of these fractions showed significant defecation-promoting effects. Nevertheless, DE18 (the fraction with the smallest fragments) clearly increased the number of black stools (*p* = 0.055 vs. model group, Figure 3E) and reduced the time for the appearance of the first black stool (*p* = 0.056 vs. model group, Figure 3G). At 1 g/kg BW (Figure 3H–L), DE12 and DE18, the two fractions with smaller fragments, significantly increased the number and weight of black stools (Figure 3J,K) while significantly decreasing the time for appearance of the first black stool (Figure 3L), with DE18 being more effective than DE12. DE12 and DE18 also increased the GI transit ratio vs. the model group (*p* = 0.053 for DE12 and *p* = 0.096 for DE18) (Figure 3H,I). DE4 and DE8 had discernible but non-significant effects compared to the model group.

Taken together, these data indicate that (1) enzymatic treatment successfully converted the ineffective pectin polymer P130D into effective fragments, and (2) within the Mw range from 0.5 to 30 kDa, the Mw of the fragments was inversely correlated with their activity. The most efficacious fraction DE18 comprised mainly dimers, trimers, tetramers, and pentamers, as determined by HPAEC (Figure S4).

Although this degradation strategy generated fragments with well-defined Mws ideal for correlation analysis, the yield of the fragments was low, especially for the most active fraction DE18. The main reason for the low yield is that this process involves a desalting step. The optimum pH of the enzyme pectinase used in this study is 3.3–4, whereas the aqueous solution of P130D (a de-esterified pectin) is neutral and thus needs pH adjustment with an acetate and acetic acid buffer, resulting in a high concentration of salt. Considering potential industrial applications, the desalting procedure used here was accomplished by employing hollow fibers with appropriate Mw cut-offs (10 kDa for fractions DE4 and DE8, 1 kDa for fractions DE12 and DE18) [37]. This step inevitably leads to low yields, especially for the fraction DE18 that contains small oligo-galacturonides. Therefore, subsequent studies should aim to explore an alternative degradation approach to bypass the desalting step.

### 3.5. Degradation of P130 and Generation of Fragments E1, E2, and E3

In an attempt to establish a simpler and higher-yield transformation process, we enzymatically hydrolyzed P130, an esterified pectin polymer, with 20% DM. Its aqueous solution has a pH of ~ pH 2.5 that is close to the working pH of the enzyme pectinase. Without pH adjustment, the desalting step (the main reason for low yields) would be omitted. Because methyl ester groups hinder the binding of pectinase to the substrate [38], using the same enzyme-to-substrate ratio as for P130D and varying the hydrolysis time would require considerably longer reaction times. Therefore, for the hydrolysis of P130, we opted to fix the reaction time and instead used different enzyme-to-substrate ratios to produce the desired fragments.

To screen hydrolytic conditions, we treated P130 with pectinase at 50 °C for 10 h over a range of enzyme-to-substrate ratios from 0.22 to 7.04 mU/g (Figure S2C). HPGPC analysis of the hydrolysates showed a clear trend of Mw reduction with increasing enzyme dosage, as evidenced by a progressive shift in the elution peaks to longer retention times (Figure S2D). Specifically, at a ratio of 0.44 mU/g, pectin degradation was clearly observed. At a ratio of 0.88 mU/g, the product fell below 30 kDa but exhibited a broad distribution. Increasing the ratio to 1.76 mU/g resulted in a distinct peak that eluted after 14 min, corresponding to a Mw range of 1 to 5 kDa. Further increasing the ratio to 3.52 mU/g produced fragments predominantly <1 kDa. At the highest ratio tested (7.04 mU/g), an elution peak appeared at 15.2 min that corresponded to the elution time of monomeric GalA.

**Figure 4:**
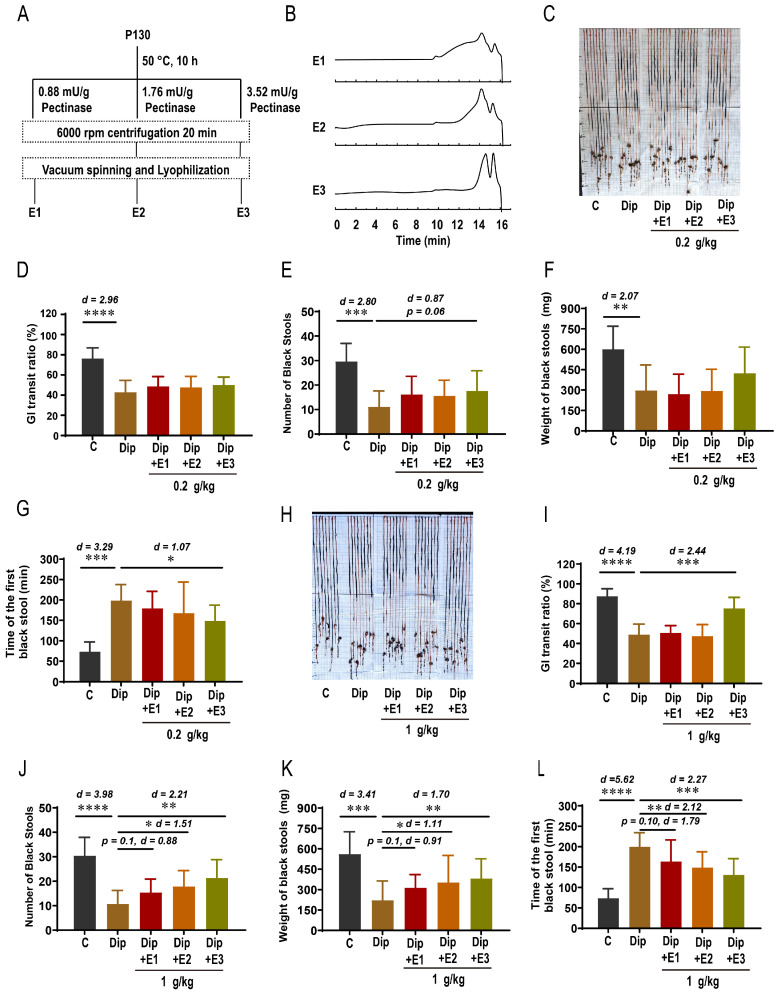
Preparation and defecation-promoting effects of pectin fragments E1, E2, and E3 from P130. (**A**) Scheme for preparation of E series fragments. (**B**) Mw distribution profiles of E series fragments. (**C**–**G**) Effects of low-dose (0.2 g/kg) fraction Es: a picture of the small intestine (**C**), the GI transit ratio (**D**), the number and weight of black stools within 5 h (**E**,**F**), and the time of appearance of the first black stool (**G**). (**H**–**L**) Effects of high-dose (1 g/kg) fraction Es: a picture of the small intestine (**H**), the GI transit ratio (**I**), the number and weight of black stools within 5 h (**J**,**K**), and the time of appearance of the first black stool (**L**). C: control group. Dip: diphenoxylate. GI: gastrointestinal. Data are shown as the mean ± SD (*n* = 8). * *p* < 0.05, ** *p* < 0.01, *** *p* < 0.001, **** *p* < 0.0001. Effect sizes were interpreted according to Cohen’s conventions (*d* ≈ 0.2, 0.5, and ≥0.8 for small, medium, and large effects, respectively).

To obtain fragments in the Mw range comparable to that of the DE series, we selected three enzyme-to-substrate ratios (0.88, 1.76, and 3.52 mU/g) for scale-up production (Figure 4A) of the obtained fractions E1 (85.2%), E2 (88.9%), and E3 (89.5%) (Table 1), respectively. Mw analysis by HPGPC showed that E1 was 5 to 30 kDa, E2 was 1 to 5 kDa, and E3 was <1 kDa (Figure 4B, Table 1). Monosaccharide composition analysis showed that all of these fragments mainly contained GalA (90.2% for E1, 95.2% for E2, and 93.1% for E3) with a small amount of Rha, Gal, and Glc residues (Table 1). The DM of all of these fractions was ~10%, slightly lower than that of the parental pectin P130 (Table 1, Figure S3B). This is likely due to the presence of some methyl de-esterification activity present in the pectinase sample used for preparation of these fragments.

Thus, we obtained a second series of fragments ≤ 30 kDa. Although the size homogeneity of this E series was not as good as that of the DE series, its production was much simpler, and the yield was much higher.

### 3.6. Effect of Fractions E1, E2, and E3 on Constipation

Subsequently, we assessed the activities of E1, E2, and E3. Again, two dose groups were set up for each sample. At a dose of 0.2 g/kg BW (Figure 4C–G), the shortest fragments fraction, E3, reduced the appearance time of the first black stool (Figure 4G), whereas other fractions had no effect. At the dose of 1 g/kg BW (Figure 4H–L), E3 significantly increased the GI transit ratio (Figure 4H,I) and the number and weight of black stools (Figure 4J,K) while decreasing the appearance time of the first black stool (Figure 4L), compared to the model group. With the medium-sized fragments, E2 also significantly increased the number and weight of black stools and decreased the appearance time of the first black stool, although it was less effective than fraction E3 (Figure 4H–L). The effect of E1 on these parameters was observable but not significant (Figure 4H–L). These results indicate that fragment size is pivotal, with shorter fragments exerting better defecation-promoting effects, a conclusion consistent with that made from the DE series fragments.

### 3.7. Fraction E3 Is More Effective than Fraction DE18

**Figure 5:**
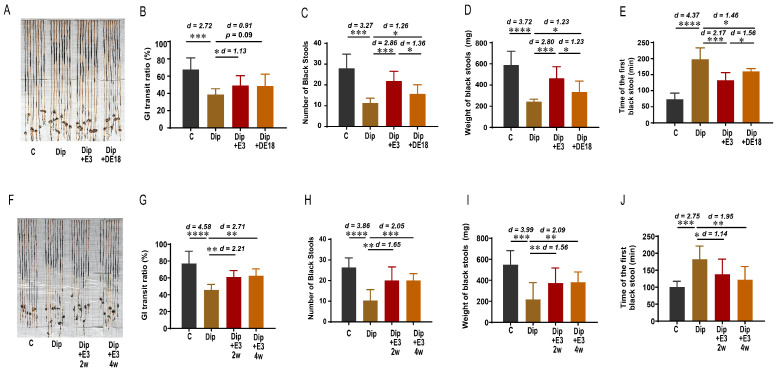
Constipation-alleviating activity of E3 and DE18. (**A**–**E**) Comparison of E3 and DE18 (1 g/kg): a picture of the small intestines (**A**), the GI transit ratio (**B**), the number and weight of black stools within 5 h (**C**,**D**), and the time of appearance of the first black stool (**E**). (**F**–**J**) Effect of E3 (1 g/kg) intervention for two (2w) and four (4w) weeks: a picture of the small intestines (**F**), the GI transit ratio (**G**), the number and weight of black stools within 5 h (**H**,**I**), and the time of appearance of the first black stool (**J**). C: control group. Dip: diphenoxylate. 2w: two-week interventions. 4w: four-week interventions. DP: degree of polymerization. GI: gastrointestinal. Data are shown as the mean ± SD (*n* = 8). * *p* < 0.05, ** *p* < 0.01, *** *p* < 0.001, **** *p* < 0.0001. Effect sizes were interpreted according to Cohen’s conventions (*d* ≈ 0.2, 0.5, and ≥0.8 for small, medium, and large effects, respectively).

**Figure 6:**
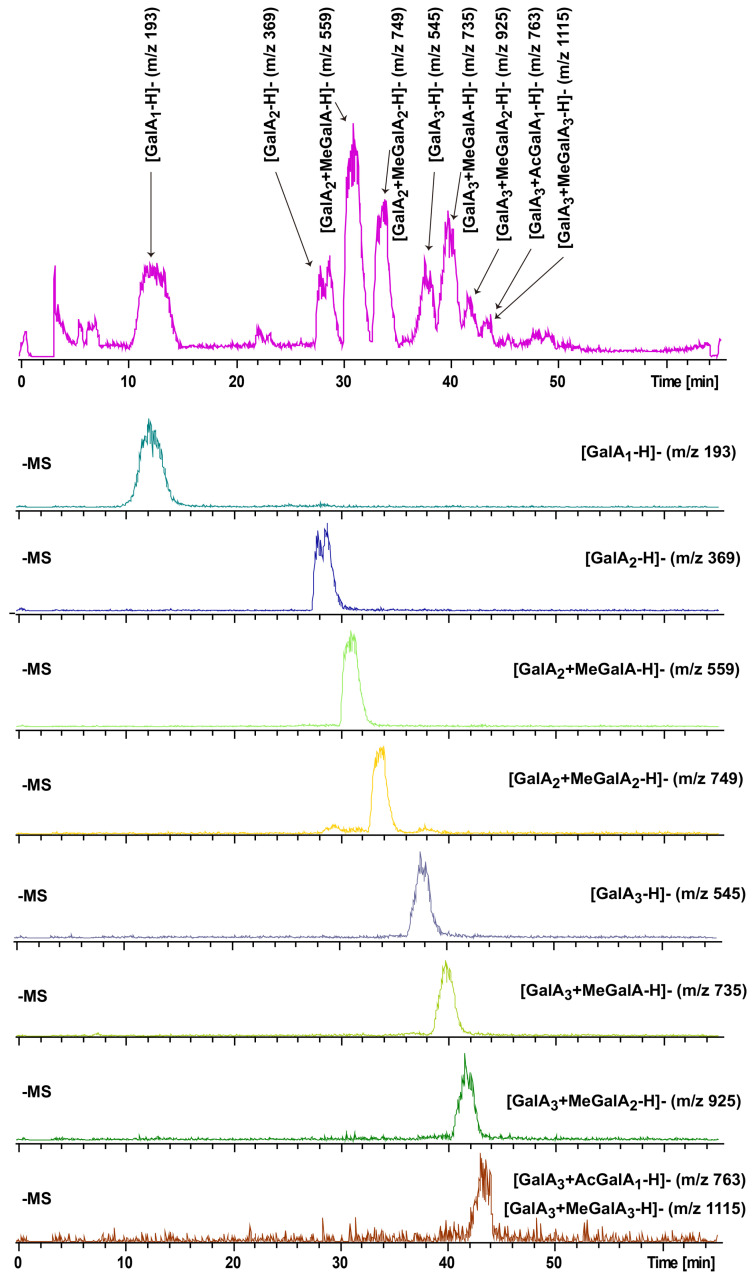
Total ion chromatogram (TIC) and extracted ion chromatograms (EICs) of oligogalacturonides in fraction E3 by UPLC-MS/MS in negative ion mode. In this mode, oligogalacturonides exist as deprotonated ions [M-H]^−^.

Results of the E and DE series show that the shortest fragments (E3 and DE18) were the most effective ones. For better comparison of their effects, we assessed E3 and DE18 simultaneously (Figure 5A–E). Clearly, E3 displays a greater effect, especially in terms of the number and weight of black stools within 5 h (Figure 5C,D) and the time of appearance of the first black stool (Figure 5E)—metrics that are significantly improved over DE18. Since conventional transit and fecal output metrics only reflect prokinetic gut acceleration, we also measured fecal water content to verify whether E3 produces authentic defecation-promoting effects via the promotion of colonic water retention to soften hardened stool. We found that E3 significantly raised fecal water content against the model control, which yielded a stronger stool-hydration improvement relative to DE18 (Figure S5). Given that E3 and DE18 have similar Mws (both <1 kDa) but different DMs (~10% for E3 and none for DE18), the greater activity of E3 suggests that esterification is another factor that governs efficacy in alleviating constipation. As methyl esterification can alter physicochemical properties and fermentation kinetics of oligogalacturonides in the gut [39], the presence of methyl esters in E3 may account for its superior effect. It should also be noted that the preparation approaches used to acquire E3 and DE18 differed, and this may result in slight differences in the fine oligomer distribution (DP ranges: E3 spans DP 1–6 and is dominated by DP3 and DP4; DE18 covers DP 2–5, with DP3 and DP4 as major fractions) (Figure 6 and Figure S4). Therefore, the greater activity of E3 over DE18 cannot be exclusively attributed to the presence of methyl ester groups. Further studies using more homogeneous oligomers would be needed to dissect the individual contributions of DP distribution and esterification to the defecation-promoting activity. Nevertheless, the aim of this study was to establish a simple method to produce pectic samples with good constipation-alleviating activities, and our results demonstrate that E3, obtained via this straightforward approach, is highly effective.

Thus far, we have identified E3 as the most effective pectin fragment. To gain more insight into this fraction, we compared two- and four-week interventions. As shown in Figure 4F–J, the activity of the four-week intervention was slightly greater than that from two-week intervention. Thus, the efficacy of E3 may be time-dependent, potentially due to progressive modulation of the gut microbial community [40].

Overall, we established a straightforward and efficient approach to convert an ineffective sunflower pectin into an effective pectic fraction. Our method employs a food-grade pectin (P130) and an easily controllable mild reaction (50 °C for 10 h) that simplifies production for a high yield (89.5%).

### 3.8. Structural Analysis of the Oligosaccharide Constituents in the Most Active Fraction E3

To elucidate the structural basis for E3 activity, we analyzed its chemical composition. UPLC-MS/MS revealed that E3 comprises oligogalacturonides, with DP ranging from one to six (Figure 6), including a monomer [GalA_1_-H]^−^ (m/z 193), dimer [GalA_2_-H]^−^ (m/z 369), trimer [GalA_3_-H]^−^ (m/z 545), mon-methyl-esterified trimer [GalA_2_+MeGalA-H]^−^ (m/z 559), mono methyl-esterified tetramer [GalA_3_+MeGalA-H]^−^ (m/z 735), di-methyl-esterified tetramer [GalA_2_+MeGalA_2_-H]^−^ (m/z 749), di-methyl-esterified pentamer [GalA_3_+MeGalA_2_-H]^−^ (m/z 925), tri-methyl-esterified hexamer [GalA_3_+MeGalA_3_-H]^−^ (m/z 1115), and mono-acetylated tetramer [GalA_3_+AcGalA_1_-H]^−^ (m/z 763). Relative abundances of these oligogalacturonides were calculated from the peak areas of EICs (Figure 6 and Table 2). Results indicated that E3 primarily comprises trimers (34.70%) and tetramers (29.24%). The monomer accounted for 20.97% and dimer for 10.98%. The pentamer (2.43%) and hexamer (1.67%) each represented a small fraction.

Tandem mass spectrometry (MS/MS) was employed to further elucidate the structures of these oligogalacturonides. The corresponding MS/MS spectra and proposed structures of the oligogalacturonides represented by these EICs peaks are shown in Figure S6 and Table 2, with fragmentation patterns annotated according to the nomenclature proposed by Domon and Costello [41]. By combining the glycosidic bond cleavage ions, the positions of methyl ester and acetyl substituents could be assigned. For mono-methyl-esterified trimer [GalA_2_+MeGalA-H]^−^ (m/z 559), diagnostic glycosidic cleavage fragments including m/z 383 [M-176-H]^−^, m/z 365 [M-194-H]^−^ were observed in its MS/MS spectrum. These ions confirm that the structure is α-D-GalA*p*-(1→4)-α-D-MeGalA*p*-(1→4)-α/β-D-GalA*p*, indicating that the methyl ester group is located on the GalA residue adjacent to the non-reducing end residue. For the mono-methyl-esterified tetramer [GalA_3_+MeGalA-H]^−^ (m/z 735), diagnostic glycosidic cleavage fragments m/z 559 [M-176-H]^−^, m/z 541 [M-194-H]^−^, m/z 369 [M-190-176-H]^−^, and m/z 365 [M-194-176-H]^−^ demonstrated that the non-reducing or reducing end residue is not methyl-esterified, whereas the residue adjacent to it carries the methyl ester group. Because the exact location of the reducing end residue could not be determined based on this analysis, the structure of this oligogalacturonide is proposed as either α-D-GalA*p*-(1→4)-α-D-GalA*p*-(1→4)-α-D-MeGalA*p*-(1→4)-α/β-D-GalA*p* or α-D-GalA*p*-(1→4)-α-D-MeGalA*p*-α-D-GalA*p*-(1→4)-α/β-D-GalA*p*. Using an analogous approach, the structure of di-methyl-esterified tetramer [GalA_2_+MeGalA_2_-H]^−^ (m/z 749) was assigned as α-D-GalA*p*-(1→4)-α-D-MeGalA*p*-α-D-MeGalA*p*-(1→4)-α/β-D-GalA*p*. For di-methyl-esterified pentamer [GalA_3_+MeGalA_2_-H]^−^ (m/z 925), two possible structures are proposed, α-D-GalA*p*-(1→4)-α-D-GalA*p*-(1→4)-α-D-MeGalA*p*-(1→4)-α-D-MeGalA*p*-(1→4)-α/β-D-GalA*p* and α-D-GalA*p*-(1→4)-α-D-MeGalA*p*-(1→4)-α-D-MeGalA*p*-(1→4)-α-D-GalA*p*-(1→4)-α/β-D-GalA*p*. In both pentamers, the non-reducing and reducing end residues lack methyl esterification, and the internal residues feature consecutively distributed methyl ester groups. For the tri-methyl-esterified hexamer [GalA_3_+MeGalA_3_-H]^−^ (m/z 1115), the proposed structure is either α-D-GalA*p*-(1→4)-α-D-MeGalA*p*-(1→4)-α-D-MeGalA*p*-(1→4)-α-D-GalA*p*-(1→4)-α-D-MeGalA*p*-(1→4)-α/β-D-GalA*p* or α-D-GalA*p*-(1→4)-α-D-MeGalA*p*-(1→4)-α-D-GalA*p*-(1→4)-α-D-MeGalA*p*-(1→4)-α-D-MeGalA*p*-(1→4)-α/β-D-GalA*p*. Again, the non-reducing and reducing end residues are devoid of methyl ester groups, while the internal residues contain two consecutive methyl esters and one separated (non-adjacent) methyl ester. For the mono-acetylated tetramer [GalA_3_+AcGalA_1_-H]^−^ (m/z 763), the MS/MS spectrum revealed fragment ions at m/z 587 [M-176-H]^−^, m/z 569 [M-194-H]^−^, m/z 411 [M-176-176-H]^−^, and m/z 351 [M-218-194-H]^−^. These data confirm that the acetyl group is located on the GalA residue adjacent to the non-reducing or reducing end residue. The structure is therefore proposed as either α-D-GalA*p*-(1→4)-α-D-AcGalA*p*-(1→4)-α-D-GalA*p*-(1→4)-α/β-D-GalA*p* or α-D-GalA*p*-(1→4)-α-D-GalA*p*-(1→4)-α-D-AcGalA*p*-(1→4)-α/β-D-GalA*p*. Acetyl substitution is possible at O-2 or O-3 of GalA.

Taken together, structural analysis showed that the highly active E3 fraction is primarily composed of low-DP oligogalacturonides (DP 1–6), some of which are methyl-esterified. The well-defined composition and consistent structural features provide a foundation for quality control in potential industrial production of this functional ingredient.

### 3.9. Prebiotic Effect of Fraction E3 on Gut Microbiota

**Figure 7:**
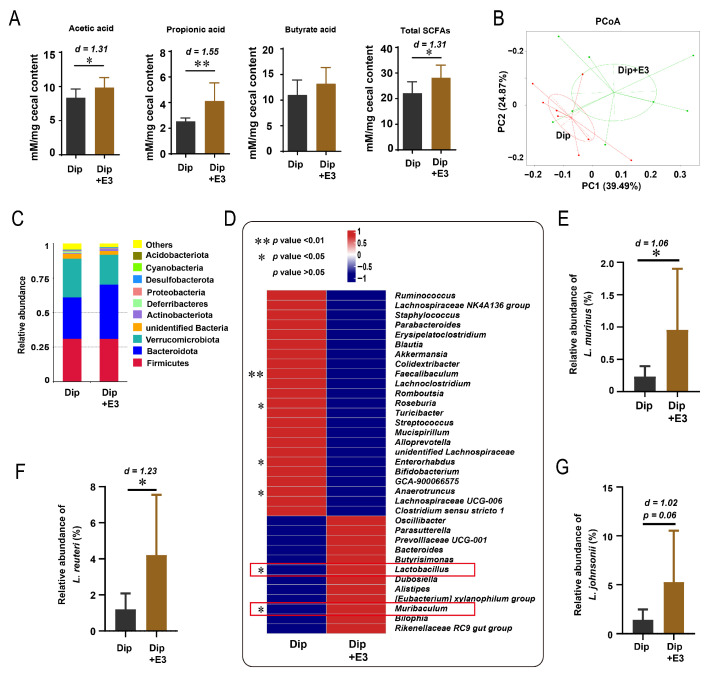
Effects of fraction E3 on SCFAs and microbial composition in cecal content in mice. (**A**) Effect of E3 on the content of SCFAs. (**B**) Principal coordinate analysis (PCoA) plots based on weighted UniFrac distance. (**C**) Relative abundance of the primary phylum in different groups. (**D**) Significance at the genus level. (**E**) Relative abundance of *Lactobacillus murinus*. (**F**) Relative abundance of *Lactobacillus reuteri*. (**G**) Relative abundance of *Lactobacillus johnsonii*. Dip: diphenoxylate. Data are shown as the mean ± SD (*n* = 8). * *p* < 0.05, ** *p* < 0.01. Effect sizes were interpreted according to Cohen’s conventions (*d* ≈ 0.2, 0.5, and ≥0.8 for small, medium, and large effects, respectively).

To further understand the activity of E3 in alleviating constipation, we investigated E3 from the perspective of gut microecology. Given that the gut microbiota influences host physiology largely through its metabolites and constipated patients often exhibit a significant down-regulation of total SCFAs [42,43], we first assessed changes in microbial fermentation products, specifically SCFAs. Gas chromatography (GC) on cecal content revealed that E3 significantly increased the concentrations of acetic acid, propionic acid, and total SCFAs compared to the constipated model control group (Figure 7A). This elevation in SCFAs is physiologically critical, because SCFAs are known to directly stimulate gut motility. Specifically, propionate has been shown to directly stimulate colonic smooth muscle contraction via GPR43 activation [44], whereas acetate serves as an energy substrate for colonocytes [45]. In addition, SCFAs can modulate serotonin (5-HT) release from enterochromaffin cells, which in turn activates enteric cholinergic neurons to promote peristalsis, thereby counteracting the opioid-induced inhibition of gut motility [46,47]. By augmenting SCFA production, E3 thus counteracts opioid-induced inhibition of intestinal motility through both direct muscular (GPR43-mediated) and indirect neural (serotonergic–cholinergic) pathways, positioning this microbial metabolic shift as a likely key mechanism underlying its laxative effect.

To trace the source of increased SCFAs, we analyzed structural changes in the gut microbiota. Principal coordinate analysis (PCoA based on weighted UniFrac distance) showed a clear separation between the E3 group and the model control group, indicating a significant shift in the overall microbial composition in the gut (Figure 7B). At the phylum level, the abundance of Bacteroidetes in the E3 group was significantly higher than that in the control group (Figure 7C). At the genus level, E3 significantly increased the relative abundance of *Lactobacillus* and *Muribaculum* (Figure 7D). Further species-level analysis revealed that E3 specifically increased the relative abundance of *Lactobacillus murinus* (*p* < 0.05, Figure 7E), *Lactobacillus reuteri* (*p* < 0.05, Figure 7F), and *Lactobacillus johnsonii* (*p* = 0.06, Figure 7G). These species are known to promote gut health and acetate/propionate production [48,49,50,51].

Notably, the concurrent enrichment of *Muribaculum*, a genus specializing in degrading complex glycans [52], and multiple *Lactobacillus* species suggests a potential functional synergy within the gut ecosystem. Interestingly, in vitro cultivation experiments revealed that *L. reuteri* and *L. johnsonii* could not grow on E3 alone, suggesting that these *Lactobacillus* species do not directly utilize oligogalacturonides. In contrast, *Muribaculum* genomes encode pectate lyases capable of degrading galacturonan-containing polysaccharides (https://www.ncbi.nlm.nih.gov/datasets/gene/GCF_001688845.2 (accessed on 20 May 2026)). Based on these observations, we hypothesize that *Muribaculum* initiates the degradation of E3’s oligogalacturonides, thus releasing metabolites that subsequently support the growth and SCFA production of *Lactobacillus* species. This cross-feeding interaction could explain the positive correlation between the enrichment of these two genera and the elevated SCFA levels observed in E3-treated mice.

From a translational perspective, the effective dose of E3 was compared with those of two established prebiotics, i.e., inulin and fructo-oligosaccharides (FOS), based on their efficacious doses in mouse constipation models. Inulin exerts complete efficacy at a dose of 2.5–5 g/kg (or 10% *w*/*w* in diet), while FOS requires 2.5 g/kg to achieve complete relief of all constipation parameters, as a lower dose (0.8 g/kg) only improves transit and fecal output without affecting first-defecation time [53,54,55]. Under identical efficacy benchmarks, E3 at 1 g/kg produced comparable comprehensive effects. Using the FDA-recommended body surface area conversion factor of 12.3 for mouse-to-human scaling [56], these doses translate to ~12.2–24.4 g/day for inulin, 12.2 g/day for FOS, and 4.9 g/day for E3 in a 60 kg adult. Thus, E3 achieved a comparable constipation-relieving efficacy at an advantageously lower dose in humans than both inulin and fully efficacious FOS. Coupled with its simple and scalable enzymatic production, E3 represents a promising functional food ingredient for constipation management. Nevertheless, clinical studies are required to confirm its efficacy and optimal dosage in humans.

In addition, from a food formulation perspective, the low-molecular-weight oligogalacturonides in E3 (predominantly DP3 and DP4) are expected to exhibit favorable physicochemical stability. Benefiting from their short chain length, these oligomers are strongly resistance to thermal degradation (e.g., β-eliminative) [57]. Moreover, their stability across a wide pH range (2.5–7) and during thermal processing suggests suitability for incorporation into various food matrices, including beverages, dairy products, and baked goods.

There are several limitations to this study. Firstly, the enzymatic hydrolysis conditions for generating E3 were optimized empirically based on time-dependent Mw profiles. In this regard, establishing a rigorous kinetic model that predicts the yield of specific oligogalacturonides as a function of enzyme and substrate concentration, as well as reaction time, would provide a valuable framework for industrial scale-up. Secondly, although E3 and DE18 have comparable Mws (<1 kDa), their preparation approaches differ, and this may result in slight differences in DP distribution. Therefore, the superior activity of E3 cannot be exclusively attributed to esterification alone. These considerations represent important directions for future research.

## 4. Conclusions

In this study, we found that native sunflower pectins by themselves have no or only minimal defecation-promoting activities, but they can be activated by degradation into fragments of lower Mw fragments, i.e., <30 kDa. Moreover, we demonstrated that the defecation-promoting effect progressively increased as the Mw decreased from 30 to 0.5 kDa, a trend that was observed with both esterified and non-esterified fragments. The most effective fraction, E3, comprised oligogalacturonides with degrees of polymerization from one to six, mainly DP3 and DP4. E3 displays prebiotic effects and can be produced easily and efficiently. Overall, our work reveals insight into the correlation between Mw and defecation-promoting activity, and establishes a simple and scalable method to convert ineffective pectin polymers into effective pectic oligogalacturonides. Given that sunflower pectin is extracted from sunflower heads, a by-product generated during oil processing, our study will be valuable in transforming residual biomass into a high-value, well-characterized functional food ingredient.

## Figures and Tables

**Table 1:** Structural features of sunflower pectin polymers and their derived fragments.

Pectins/	Yield	DM ^a^	Mw ^b^	Monosaccharide Composition (%)				
Fragments	(%)	(%)	(kDa)	GalA	Rha	Gal	Glc	Ara
P30	7.4 ^c^	25	30	86.2	2.0	2.3	1.9	7.6
P75	7.5 ^c^	30	75	85.2	2.3	3.5	3.7	5.3
P130 ^d^	-	20	130	93.0	2.9	2.2	0.9	1.0
P30D	90.5 ^e^	0	30	89.0	2.0	4.2	3.8	1.0
P75D	93.3 ^e^	0	75	94.3	2.0	2.0	0.8	0.9
P130D	96.7 ^e^	0	130	94.1	3.6	1.6	0.7	bl
DE4	78.4 ^f^	0	30	96.0	2.0	1.5	0.5	bl
DE8	48.9 ^f^	0	15	94.7	3.6	1.2	0.5	bl
DE12	20.1 ^f^	0	4	91.1	5.8	2.1	1.0	bl
DE18	9.0 ^f^	0	<1	96.4	1.8	1.1	0.7	bl
E1	85.2 ^g^	10	5–30	90.2	2.3	3.4	2.7	1.4
E2	88.9 ^g^	10	1–5	95.2	2.0	1.2	1.1	0.5
E3	89.5 ^g^	10	<1	93.1	2.2	2.0	1.4	1.3

Note: a—degree of methyl esterification (DM); b—weight-average molecular weight (Mw); c—extraction yield, calculated by using Equation (1), relative to the dry weight of the sunflower head; d—commercial product; e—yield, calculated by using Equation (2), relative to the weight of the parent esterified pectin; f—yield, calculated by using Equation (3), relative to the weight of the parent P130D pectin; g—yield, calculated by using Equation (4), relative to the weight of the parent P130 pectin; bl—below detection limit.

**Table 2:** Putative structures and relative abundance of oligogalacturonides in fraction E3 assigned by UPLC-MS/MS.

Elution Time (Min)	m/z	DP ^1^	Relative Abundance (%)	Structural Constitution
12.8	193	1	20.97	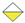
29.2	369	2	10.98	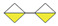
37.3	545	3	7.58	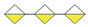
30.8	559	3	27.13	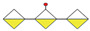
39.8	735	4	11.95	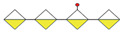 or 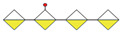
30.3	749	4	17.29	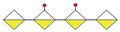
43.1	763	4	1.67 ^2^	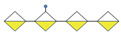 or 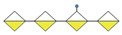
41.8	925	5	2.43	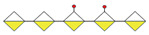 or 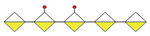
43.1	1115	6	1.67 ^2^	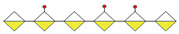 or 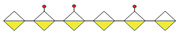

^1^ DP: degree of polymerization. ^2^ The m/z 763 and m/z 1115 species have a collective relative abundance of 1.67%. 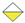: galacturonic acid. 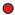: methyl ester. 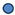: acetyl.

## Data Availability

The original contributions presented in this study are included in the article/Supplementary Materials. Further inquiries can be directed to the corresponding authors.

## Supplementary Materials

The following supporting information can be downloaded at: https://www.mdpi.com/article/10.3390/foods15152625/s1, Figure S1: Assessment of constipation-alleviating activity of P130D, P75D and P30D.; Figure S2: Screening for enzymatic hydrolysis conditions; Figure S3: FT-IR analysis of pectin fragments E3 and DE18; Figure S4: HPEAC analysis of DE18; Figure S5: Fecal water content in each group; Figure S6: Extracted ion chromatograms (EICs), MS^2^ spectra, and proposed structures of the oligogalacturonides; Table S1: Cohen’s d and 95% CI for intergroup comparisons.

## Abbreviations

The following abbreviations are used in this manuscript:

ABTS	2,2′-azino-bis (3-ethylbenzothiazoline-6-sulfonic acid)
ANOVA	Analysis of variance
Ara	Arabinose
ASV	Amplicon sequence variant
BLAST	Basic local alignment search tool
DCM	Dichloromethane
Dip	Diphenoxylate
DM	Degree of methyl esterification
DP	Degree of polymerization
DPPH	2,2-diphenyl-1-picrylhydrazyl
FT-IR	Fourier-transform infrared spectroscopy
Gal	Galactose
GalA	Galacturonic acid
GC	Gas chromatography
GI	Gastrointestinal
Glc	Glucose
HG	Homogalacturonan
HPLC	High-performance liquid chromatography
HPGPC	High-performance gel permeation chromatography
LCA	Lowest common ancestor
PMP	1-phenyl-3-methyl-5-pyrazolone
Rha	Rhamnose
SCFA	Short-chain fatty acid
