# Supplementary material for: Sunflower-Derived Pectin Oligogalacturonides Promote Defecation in Mice
Source: Foods. 2026 Jul 27;15(15):2625. doi: 10.3390/foods15152625 (PMC13463959; doi:10.3390/foods15152625)
Supplement: Supplementary file 1 [file foods-15-02625-s001.zip › Supplementary materials.pdf]

Supplementary materials

Sunflower-derived pectin oligogalacturonides promote defeca-tion in mice

Hongming Gu [1], Xian Qu [3], Xiaotong Sheng [1], Xuran Wang [1], Yuhan Cai [1], Jie Geng[1], Liangnan Cui [1], Kevin H. Mayo [2], Yifa Zhou [1], Hairong Cheng [1*], Guihua Tai [1*]

[1] Engineering Research Center of Glycoconjugates, Ministry of Education, Jilin Provincial Key Laboratory of Chemistry and Biology of Changbai Mountain Natural Drugs, School of Life Sciences, Northeast Normal University, Changchun, 130024, China
[2] Department of Biochemistry, Molecular Biology & Biophysics, 6-155 Jackson Hall, University of Minnesota, 321 Church Street, Minneapolis, MN, 55455, USA
[3] State Key Laboratory of Cellular Stress Biology, Faculty of Medicine and Life Sciences, School of Life Sciences, Xiamen University, Xiamen 361000, China
* Correspondence: taigh477@nenu.edu.cn (GH Tai); chenghr893@nenu.edu.cn (HR Cheng); Tel.: +86-431 85099165 (GH Tai)

1. Method

1.1 Galacturonic acid oligosaccharides analysis by HPAEC

Oligosaccharides were dissolved in deionized water (20 μg/ml) and separated on a CarboPac PA200 column (3 × 250 mm) using the High-Performance Anion-Exchange Chromatography (HPEAC) system. The column was eluted at 0.5 ml/min with the following gradient: isocratic elution with 30 mM NaAc/100 mM NaOH from -10 to 0 min; linear gradient from 30 to 250 mM NaAc/100 mM NaOH from 0.01 to 20 min; then isocratic elution with 500 mM NaAc/200 mM NaOH from 20.01 to 35 min.

1.2 Determination of Fecal Water Content

Fresh fecal pellets were collected from each mouse immediately after defecation and transferred into pre-weighed sterile polyethylene tubes. The wet weight of the feces was recorded promptly. Subsequently, the tubes were placed in a forced-air oven and dried at 55°C for 24 hours (or until a constant weight was achieved, as confirmed by successive weighings). After drying, the tubes were cooled to room temperature in a desiccator to prevent moisture reabsorption, and the dry weight was measured. The moisture content was calculated as the

percentage of weight loss relative to the initial wet weight.

2. List of Figures

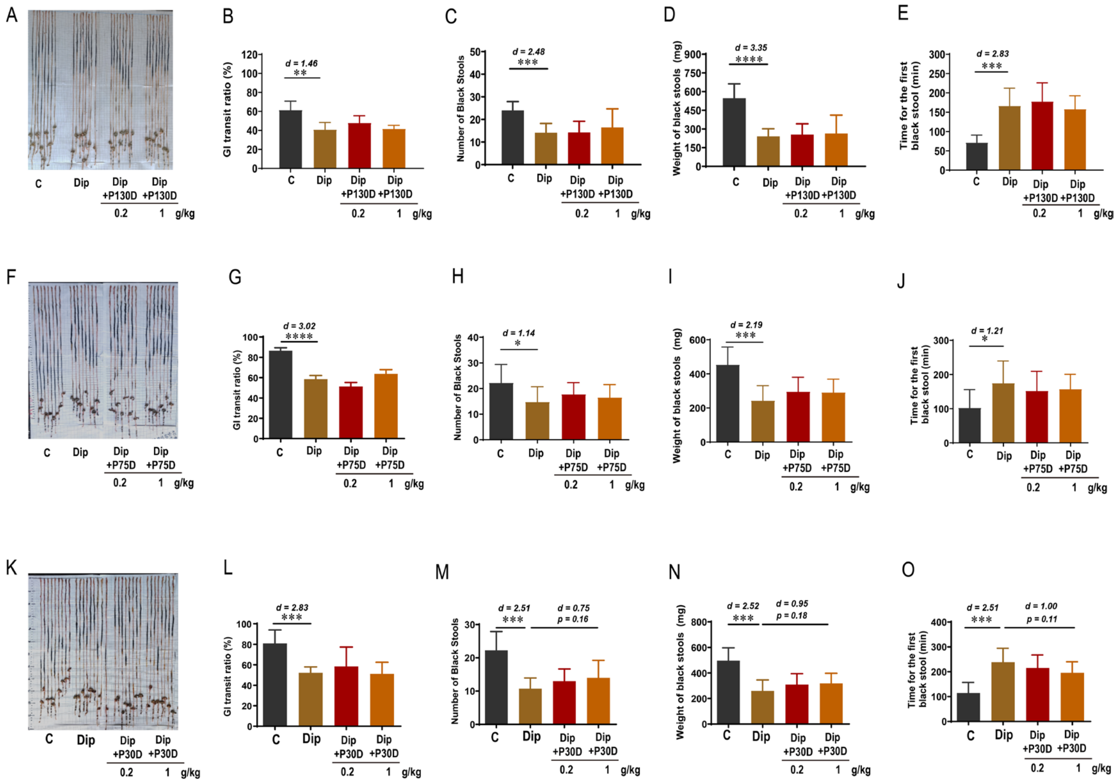

Figure S1. Assessment of constipation-alleviating activity of P130D, P75D and P30D. (**A-E**) Constipation-alleviating activity of high-dose and low-dose P130D: picture of the small intestine (**A**), GI transit ratio (**B**), number and weight of black stool within 5 hours (**C, D**), and appearance time for the first black stool (**E**). (**F-J**) Constipation-alleviating activity of high- and low-dose P75D: picture of small intestine (**F**), GI transit ratio (**G**), number and weight of black stool within 5 hours (**H, I**), and appearance time for the first black stool (**J**). (**K-O**) Effects of high- and low-dose P30D: picture of the small intestine (**K**), GI transit ratio (**L**), number and weight of black stool within 5 hours (**M, N**), and appearance time for the first black stool (**O**). C: Control group. Dip: Diphenoxylate. Data are shown as the mean ± SD (n=8). *$p < 0.05$, **$p < 0.01$, ***$p < 0.001$, ****$p < 0.001$. Effect sizes were interpreted according to Cohen's conventions ($d \approx 0.2$, 0.5, and ≥ 0.8 for small, medium, and large effects, respectively).

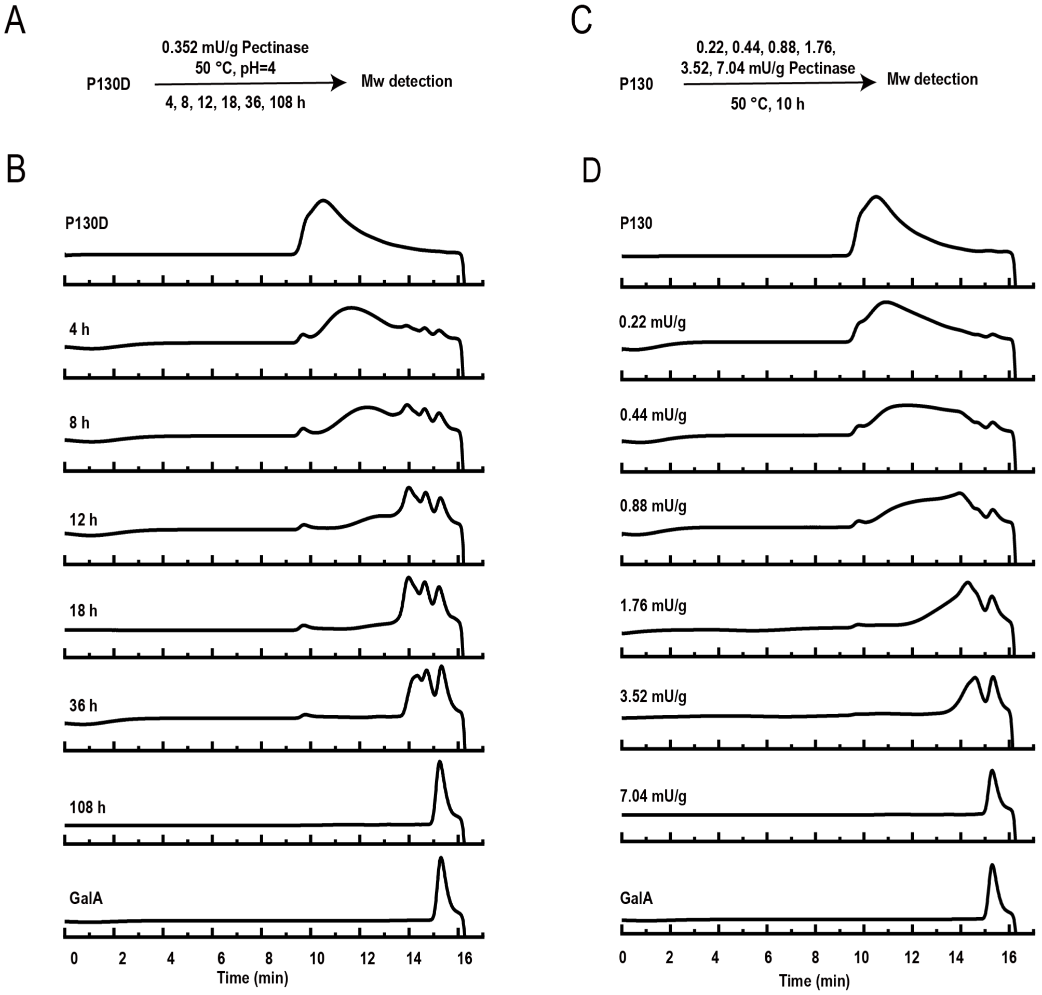

Figure S2. Screening for enzymatic hydrolysis conditions. (**A**) Scheme for hydrolysis of P130D. (**B**) Mw distribution profiles of P130D after hydrolysis for different time periods. (**C**) Scheme for pectinase hydrolysis of P130. (**D**) Mw distribution profiles of P130 after 10 h hydrolysis at different pectinase concentrations.

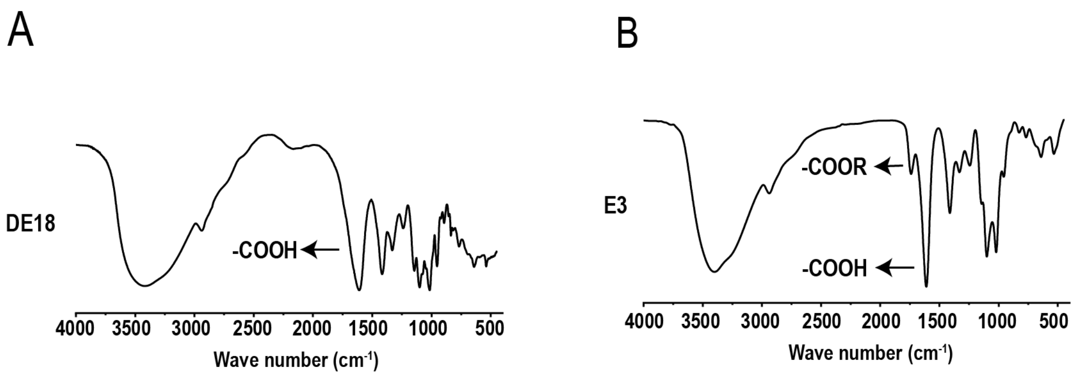

Figure S3. FT-IR analysis of pectin fragments E3 and DE18. (**A**) FT-IR spectrum of E3. (**B**) FT-IR spectrum of DE18.

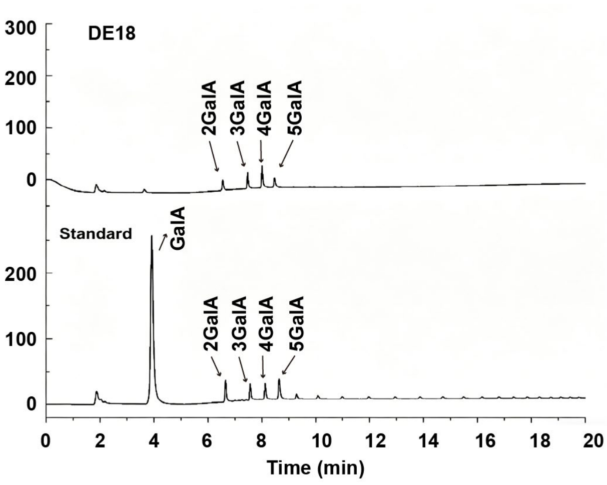

Figure S4. HPEAC analysis of DE18.

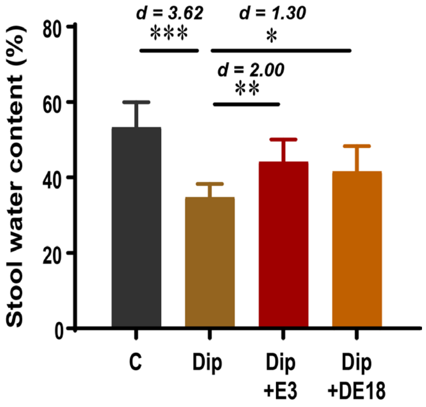

Figure S5. Fecal water content in each group. C: Control group. Dip: Diphenoxylate. Data are shown as the mean ± SD (n=8). *$p < 0.05$, **$p < 0.01$, ***$p < 0.001$. Effect sizes were interpreted according to Cohen's conventions ($d \approx 0.2$, 0.5, and ≥ 0.8 for small, medium, and large effects, respectively).

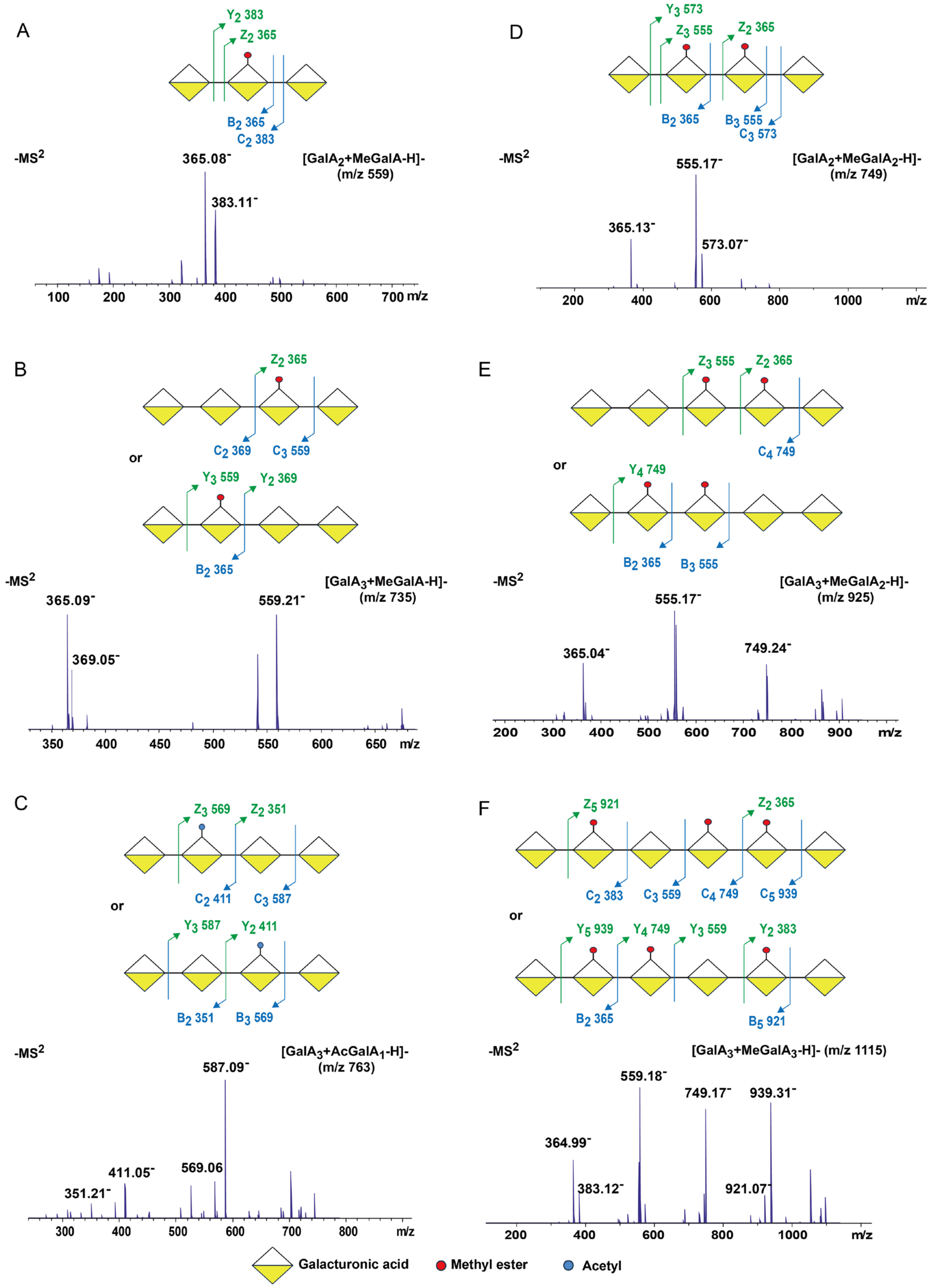

Figure S6. Extracted ion chromatograms (EICs), MS^2 spectra, and proposed structures of the oligogalacturonides.
